# The Standardization of Medical Examining Boards

**Published:** 1905-01

**Authors:** 


### THE STANDARDIZATION OF MEDICAL EXAMINING BOARDS.

THE need for State examining boards to get together on some reciprocal basis has been growing for a long time. But there have been and are obstacles. In neighboring and adjoining States where the standard of medical education, provided there be a medical college within the boundary of each, is practically on a par, and where the practice of frontier physicians is not restricted by State or geographical lines, reciprocity is eminently wise, convenient and fitting. But in the instances where the States are widely separated, where the qualifications for graduation are high in one, low in another, is reciprocity altogether proper and advisable? In certain States medical education is exceedingly advanced, the public demands thoroughly competent and well-educated physicians; in another State the population is largely rural, the inhabitants healthy as a whole, and are content to have their pathological deviations corrected by one to whom the term "Doc" is befitting. It is hardly necessary to say that "Doc" would bite the dust in a tilt with examiners for whom the better equipped man would have no fear. To get around this lack of uniformity in the character of examination and the degree of proficiency required in the applicant by the different boards, the suggestion has been made that the examinations be conducted by a board appointed by the government at Washington, the members selected from the medical personnel of the army. This board would be entirely non-partisan and it would make no difference whether the applicant hailed from Florida or Maine, Oregon or Georgia, so long as he proved his ability by the test of thorough examination. Having passed this board he would be in possession of a sort of roving commission that would enable him to ply his avocation wherever his fancy led him without having to take on another license from a State board. This plan would work something of a hardship if enforced upon the older graduate, whose knowledge of the rudiments of medicine as embraced in chemistry, anatomy and the like was somewhat dimmed by the mist of years, but it would be possible to take care of him under the years of practice act or in some such manner, as is at present in vogue in some States. These veterans would, in the process of time, be weeded out and in the end all physicians could be on a parity in the mode of obtaining license.

The suggestion is assuredly worth considering as a way out of the present very unsatisfactory state of affairs.

---

THE number of registered physicians in the Russian empire, according to an exchange, is now 21,827, of whom 737 are women. All but 2,659 reside in European Russia. Siberia has 788, the Caucasus district 1,224, and the Central Asia possessions 459. The last restrictions have been removed from the medical career for women, and the graduates of the woman's medical college at St. Petersburg now rank in every respect with the graduates of the other medical schools of the empire.

---

THE new Vienna hospital, of which the foundation stone was recently laid by the Emperor of Austria, will consist of forty separate buildings, eight of which will be devoted to offices and residences for the staff. Each of the clinical buildings will have a roof-garden, and each patient will be allowed 1,030 square feet of space. The hospital will have 2,300 beds. The operating-rooms will be of an entirely new type, and in the clinics for infectious diseases the patients will be separated from the lecturer and the students by a glass partition. The entire cost of the hospital, says an exchange, will be between $7,000,000 and $8,000,000.

